# Histopathologic patterns of intracranial neoplasms at Lagos University Teaching Hospital, Nigeria. A ten-year hospital-based retrospective study

**DOI:** 10.4314/ahs.v23i1.51

**Published:** 2023-03

**Authors:** Kasiemobi E Uchime, Luqman A Adebayo, Lateef A Odukoya, Olugbende O Ajayi, Charles C Anunobi

**Affiliations:** 1 Afe Babalola University College of Medicine and Health Sciences, Anatomic Pathology and Forensic Medicine; Lagos University Teaching Hospital, Anatomic and Molecular Pathology; 2 Lagos University Teaching Hospital, Department of Anatomic and Molecular Pathology; 3 Stellenbosch University Faculty of Medicine and Health Sciences, African Cancer Institute; Lagos University Teaching Hospital, Department of Anatomic and Molecular Pathology; 4 College of Medicine of University of Lagos, Department of Anatomic and Molecular Pathology; Lagos University Teaching Hospital, Department of Anatomic and Molecular Pathology

**Keywords:** INTRACRANIAL, Neoplasms, Central Nervous System

## Abstract

**Background:**

The most common intracranial neoplasm worldwide is meningioma, followed by gliomas, and then pituitary adenomas. There are geographical differences in the pattern of occurrence of intracranial neoplasms.

The purpose of this study is to establish the pattern of occurrence of different histological types of intracranial neoplasms with their age and sex distributions in our environment – Lagos, Nigeria.

The histological patterns, age, and gender distributions of all the intracranial neoplasms diagnosed within the study period at the Department of Anatomic and Molecular Pathology, LUTH, Lagos, Nigeria were noted and analysed with SPSS version 23.

**Result:**

There were 296 patients (165 females, 131 males; mean age of 37.0 years) diagnosed with an intracranial neoplasm within the study period. The most frequently diagnosed intracranial neoplasm was meningioma (105 cases; 35%, median age of 42 years, male to female ratio of 1:2.2), followed by pituitary adenoma (78 cases; 26%, median age of 47 years, male to female ratio of 1.3:1), and then gliomas (71 cases; 24%, median age of 28, male to female ratio of 1:1.39).

**Conclusion:**

The result of the study shows pituitary adenoma to be more common than gliomas, unlike what is seen in Caucasians where the reverse is the case.

## Introduction

Tumors of the central nervous system account for 1-2% of tumors in adults worldwide.^1^ An intracranial tumor/neoplasm (ICT/ICN), also colloquially known as a brain tumor, may arise from the brain or its covering structures (primary intracranial tumor) or may be a metastasis from another organ (metastatic intracranial tumor). Awodele *et al.*, in a study based on the Lagos and Ibadan Cancer registries in Nigeria, between 2005 and 2009, reported that brain tumors constitute 3.9% of all cancer types.^2^

Over 150 different brain tumors have been documented and they have distinct diagnostic histological features, some with distinct molecular/cytogenetic characteristics while some are common in certain gender or age groups.^3^ There are many prognostic factors that influence the clinical outcomes of patients with intracranial tumors. Some of the prognostic factors include tumor's histological grade and biologic behaviour, the patient's age at diagnosis (young patients have better prognosis), and tumor location (tumors with pressure effect on vital structures pose poorer prognosis).^4^

The most common intracranial neoplasm worldwide is meningioma, followed by gliomas, most especially astrocytic tumors, and then pituitary adenoma.^1^,^3^ There are geographical differences in the pattern of occurrence of intracranial neoplasm and increasing incidence of primary brain tumors worldwide in recent decades.^5^ The global pattern of intracranial tumors is as shown in Table 1, and it depicts meningioma and glioma as the two commonest ICT and pituitary adenoma as the 3rd most common ICT globally.^6^–^16^

**Table 1:** Global pattern of intracranial tumors

Author	Country	Total	Commonest ICT	2^nd^ most common ICT	3^rd^ most common ICT
Stoyonov **et** **al.** (Jan-Dec, 2016)^6^	Eastern Bulgaria	822 (544 are primary ICT)	Glial tumours (47.24%)	Meningioma (39.15%)	Pituitary adenoma (8.2%)
Aryal *et* *al.* (1998–2000)^7^	Nepal	57	Astrocytoma (38.6%)	Meningioma (14%)	Metastatic tumor (14%)
Nakamura *et* *al.* 2011^8^	Japan	5448	Meningioma (36.8%)	Glioma (19.5%)	Pituitary adenoma (17.8%)
Cordera *et* *al.* 2002^9^	Italy	285	Meningioma (37%)	Glioma (35%)	Pituitary adenoma (8%)
Kuiper *et al.* 2013^10^	Suriname	251	Meningioma (26.7%)	Glioma (21%)	Pituitary adenoma (12%)
Katchy *et* *al.* 2011^11^	Kuwait	822	Glioma (33%)	Meningioma (28%)	Pituitary adenoma (19%)
Surawicz *et* *al.* 1999^12^	USA	20,765	Glioma (49%)	Meningioma (24%)	Pituitary adenoma (8%)
Mehrazin *et* *al.* 2006^13^-	Iran	3437	Glioma (33.3%)	Meningioma (26%)	Pituitary adenoma (14.2%)
Anadure *et* *al.* Jan2012– Jun 2015^14^	Kalaburgi, India	50 neoplastic cases	Glioma (50%)	Schwannoma (14%)	Meningothelial tumors (12%)
Kuratsu and Ushio, 1996^15^	Japan (primary ICT in children)	2129	Meningioma (33.3%)	Glioma (19.9%)	Pituitary adenoma (18.3%)
Ekpene *et* *al.* (Jan 2010 to Dec. 2015)^16^	Ghana	102	Glioma (38.2%)	Meningioma (36.2%)	Pituitary adenoma (8.8%)

Few studies have been conducted in Nigeria to demonstrate the pattern of occurrence, age, and sex distributions of these intracranial neoplasms. The result of most of these studies carried out in Nigeria still showed meningioma as the commonest ICT. However, most of these studies showed that pituitary adenoma is rather the second most common ICT in Nigeria, (see Table 2).^17^–^23^

**Table 2:** Pattern of ICT in Nigeria

Author	Year of study	Place of Study	Total ICT	Commonest ICT	2^nd^ Most Common ICT	3^rd^ Most Common ICT
Ohaegbulam *et* *al.*^17^	Jan. 1974 to March 1979	Enugu	48	Glial tumors (20.8%)	Pituitary tumors (18.8%)	Meningioma (16.7%)
Olosode *et al.*^18^	1980–1990	Ibadan	210	Glioma (33%)	Metastatic tumors (23%)	Pitutary adenoma (17.1%) Meningioma (11.4%)
Jibrin *et al.*^19^	Jan. 2005 to Dec. 2015	Abuja	121	Meningioma (41%)	Pituitary adenoma (22%)	Glioma (20%)
Igun G. O.^20^	Jan. 1989 to Nov. 1998	Jos	30	Metastatic tumors (30%)	Anterior pituitary tumors (21%)	Meningeal tumors (18%)
Ndubuisi *et al.*^21^	2015	Enugu	252	Meningioma (32.9%)	Glioma (23.8%)	Pituitary adenoma (18.5%)
Idowu *et al.*^22^	Jan 1999 to Dec. 2004	UCH (Ibadan)	113 (All primary ICT)	Total: Glioma (23%) Among Adults: Meningioma (30%)	Total: Meningioma (23%) Among Adults: pituitary adenoma (27%)	Total: Pituitary adenoma (16.8%) Among Adults: Glioma (21.1%)
Malami *et al.*^23^	Jan. 2008 to Dec. 2017	Sokoto	151	Meningioma (37%)	Glioma (Astrocytoma) 23.3%	Pituitary tumors: Pituitary adenoma (7.3%), craniopharyngioma (11.9%)

Lagos is a densely populated fast-growing city in Nigeria and Lagos University Teaching Hospital (LUTH) is one of the largest tertiary hospitals in Lagos State, Nigeria.24 Lagos had an estimated population of 21 million in 2016, which makes it the most populous city in Africa.24 Given the dense population of Lagos state, this study will give a good representation of the health burden and pattern of occurrence of intracranial tumors in Western Nigeria. The study aims to establish the relative frequency of occurrence of biopsy-proven intracranial neoplasms at Lagos University Teaching Hospital (LUTH), Idi-Araba, Lagos, Nigeria, from the1st January 2008 to the 31st December 2017. Secondly, to determine the frequency of occurrence, age, and gender distribution of the different histological types of intracranial neoplasms according to the 2016 World Health Organization's (WHO) classification of tumors of the central nervous system. Finally, to compare our results with studies from other centers in Nigeria and worldwide.

## Material and Method

The 2016 WHO classification of tumors of the central nervous system was used to stratify all the patients diagnosed with intracranial neoplasm at the Department of Anatomic and Molecular Pathology, Lagos University Teaching Hospital (LUTH), Idi-Araba, Lagos, Nigeria within a 10-year study period. The study was a 10-year (January 1^st^, 2008- December 31^st^,2017) hospital based retrospective study.

Patients' data were retrieved from the archives of the Department of Anatomic and Molecular Pathology, LUTH, Idi-Araba, Lagos. Histological patterns of these intracranial tumors, with their grade, age and gender distribution were retrieved and recorded for analysis. Cytological smears (squash cytology) and immunohistochemistry were not carried out on these samples. Due to the old manual recording system of the medical record of the hospital, the exact locations of some of these intracranial tumors could not be obtained.

The data obtained was analysed with SPSS version 23.

Due to unavailability of facilities for appropriate molecular studies in our centre, diffuse astrocytoma, anaplastic astrocytoma, glioblastoma and oligodendroglioma were regarded as “not otherwise specified” (NOS), according to the 2016 WHO classification of intracranial tumors guidelines.

## Result

Two hundred and ninety-six patients (165 females, 131 males) were diagnosed with intracranial neoplasm/tumor (ICT) within the study period at the department (Table 3). Intracranial neoplasms constituted 1.06% of the total biopsies received at LUTH from 1st of January, 2008 to 31st of December, 2017, with a male to female ratio of 2:3(Table 3).

**Table 3:** Age and gender distribution of all biopsy-proven intracranial neoplasms at LUTH between 1^st^ of January, 2008 to 31^st^ of December, 2017

Variable	Frequency (n=296)	Percentage
**Gender**		
Male	131	44.3
Female	165	55.7
**Age group (Years)**		
≤10	36	12.2
11–20	21	7.1
21–30	30	10.1
31–40	61	20.6
41–50	70	23.6
51–60	41	13.9
61–70	29	9.8
71–80	8	2.7
**Median (Q1-Q3): 40.50** **(27.3–51.0)** **Mean: 37.0**		

**Figure 1:**
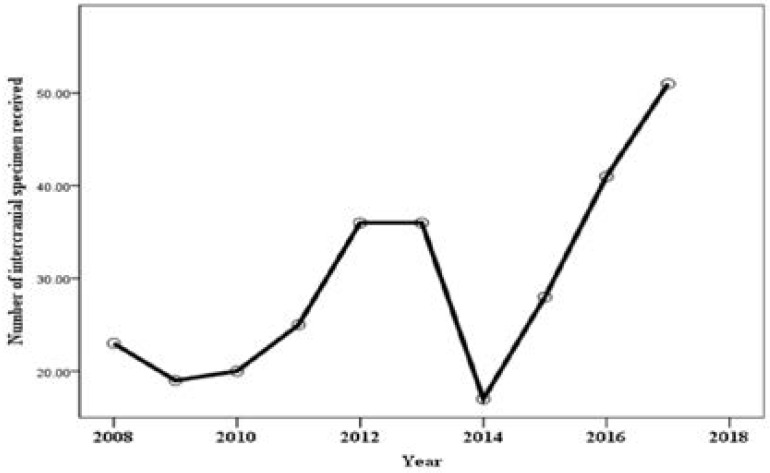
Number of intracranial specimens received yearly at Anatomic and Molecular Department, LUTH, between 1st of January, 2008 and 31st of December, 2017

Overall, the department saw the highest number ICT diagnosis in 2017 (51 cases, 17% of the total 296 cases), while 2014 had the lowest number of diagnosed cases (10 cases, 3% of the total 296 cases) (Figure 1).

**Figure 2:**
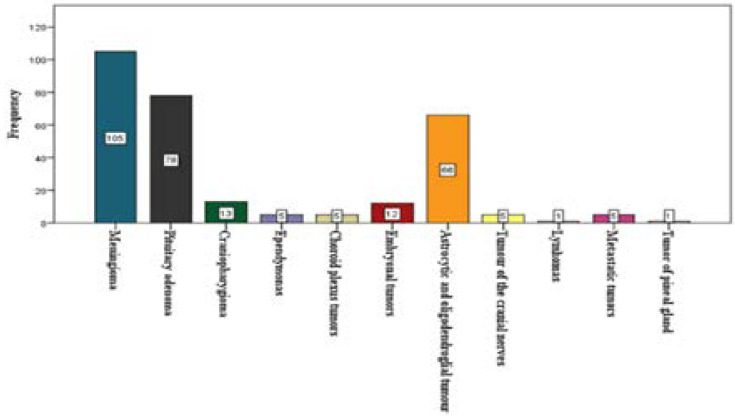
Frequency of occurrence of different biopsy-proven intracranial neoplasms at LUTH between 1st of January, 2008 and 31st of December, 2017.

Majority of the patients with intracranial neoplasm were between 41-50 years of age (23.6% of the total intracranial neoplasm) (Table 1). The median age of ICTs was 40.50 years (IQR=30.3), while the mean age of ICTs was 37.0 years (Table 3).

rimary ICTs accounted for 98.3% (291cases) of the total ICTs, whereas metastatic ICTs accounted for 1.7% (5 cases, 4 metastatic carcinoma and one metastatic osteosarcoma) of the total ICTs (Figure 2). The median age of patients with histology confirmed metastatic ICTs was 39 years (Table 4).

**Table 4:** Median age of different intracranial neoplasm diagnosed in LUTH between 1^st^ of January, 2008 and 31^st^ of December, 2017

Intracranial neoplasm	Median age	IQR (Q1, Q3)	Range
Meningioma	42.00	34.0, 54.5	1, 80
Pituitary adenoma	47.00	36.8, 50.5	15, 73
Craniopharyngioma	23.00	12.0, 41.5	2,56
Ependymoma	19.00	2.5, 46.0	1,60
Choroid plexus tumor	10.00	1.5, 44.0	1,51
Embryonal tumors	6.00	3.5., 10.8	1,21
Astrocytic and Oligodendroglia tumors	37.00	10.0,50.3	1,75
Tumor of the cranial nerves	42.00	18.0, 48.0	3, 51
Metastatic tumor	39.00	34.0, 61.0	19,68

1 subject had a tumor of pineal gland and another had lymphoma (Non-Hodgkin Lymphoma). These patients were aged 3 years and 74 years respectively.

Children aged 10 years and below constituted 12.2 % (36 cases) of the total intracranial neoplasms, majority of whom were diagnosed with pilocytic astrocytoma, embryonal tumours (including medulloblastoma) and choroid plexus tumors with male to female ratio of 1:1, 7:5 and 4:1 respectively (Tables 3, 4, & 5).

**Table 5:** Gender distribution of different intracranial neoplasm diagnosed in LUTH between 1^st^ of January, 2008 and 31^st^ of December, 2017

Diagnosis	Male (n=131)	Female (n=165)
Meningioma	33(31.4%)	72(68.6%)
Pituitary adenoma	48(61.5%)	30(38.5%)
Craniopharyngioma	4(30.8%)	9(69.2%)
Ependymomas	3(60.0%)	2(40.0%)
Choroid plexus tumor	4(80.0%)	1(20.0%)
Embryonal tumors	7(58.3%)	5(41.7%)
Astrocytic and oligodendroglia tumors	28(42.4%)	38(57.6%)
Tumor of the cranial nerves	2(40.0%)	3(60.0%)
Lymphoma	0(0.0%)	1(100.0%)
Metastatic tumors	2(40.0%)	3(60.0%)
Tumor of pineal gland	0(0.0%)	1(100.0%)

P=0.010*

The most frequently diagnosed intracranial neoplasm at LUTH within the study period was meningioma (105 cases, 35.5%, median age of 42 years with a male to female ratio of 3:7). This was followed by pituitary adenoma (78 cases, 26.4%, median age of 47 years, with a male to female ratio of 3:2), and then gliomas (71 cases; 24%, median age of 28, male to female ratio of 1:1.3), including the astrocytic and oligodendroglial tumors (66 cases, 22.3%, median age of 37 years with a male to female ratio of 2:3), and ependymoma (5 cases, 1.7%, median age of 19 years with male to female ratio of 3:2), (Figure 2, Tables 3,4&5).

Lymphomas and tumors of the pineal gland were the least diagnosed intracranial neoplasms. There was one case of pineal gland tumor and one case of lymphoma diagnosed in patients aged 3 years and 74 years respectively (Figure 2).

Amongst the gliomas, pilocytic astrocytoma (22 cases, median age of 7.5 years, male to female ratio of 1:1) and glioblastoma NOS (22 cases, median age of 56.5years, male to female ratio of 4:7) were the most frequently diagnosed. Oligodendroglioma (1 case, 26 years, female) was the least diagnosed glioma (Tables 6).

**Table 6:** Age and Sex distribution of gliomas diagnosed at LUTH, between 1^st^ of January, 2008 and 31^st^ of December, 2017

Diagnosis	Male (n=28)	Female (n=38)	Total number	Median age	Age: IQR (Q1, Q3)
Pilocytic astrocytoma	11(50.0%)	11(50.0%)	22	7.50	4.0, 15.0
Pleomorphic Xanthoastrocytoma	0(0.0%)	2(100.0%)	2	16.50	13.0, 20.0
Diffuse astrocytoma (NOS)	8(61.5%)	5(38.5%)	13	38.00	24.5, 45.5
Anaplastic astrocytoma (NOS)	1(16.7%)	5(83.3%)	6	46.50	37.3, 54.3
Glioblastoma (NOS)	11 50.0%)	11(50.0%)	22	56.5	46.8, 63.5
Ependymoma	3(60.0%)	2(40.0%)	5	19.00	2.5, 2.60

Only one patient with Oligodendroglioma (26 years, female) was diagnosed. P=0.249

Most (94.29%) of the meningioma cases diagnosed within the study period were WHO grade I; majority (55%) of them had the histological variant of meningothelial meningioma (Table 7). Only 5.7% of all the diagnosed meningioma cases were classified as WHO grade II tumors. There was no WHO grade III variant of meningioma diagnosed during the study period (Table 7).

**Table 7:** Histological pattern of meningioma cases seen within the study period – between 1^st^ of January, 2008 and 31^st^ of December, 2017t LUTH

Histologic Variants of Meningioma	Number Of Cases	Percentage Frequency
Meningothelial meningioma (grade 1)	58	55%
Transitional meningioma (grade 1)	20	19%
Fibroblastic meningioma (grade 1)	6	6%
Angiomatous meningioma (grade 1)	7	7%
Psammomatous meningioma (grade 1)	8	8%
Atypical meningioma (grade 2)	5	5%
Clear cell meningioma (grade 2)	1	1%
Total	105	100%
**There was no case of WHO grade 3**		
meningioma seen within the study period.		

Majority (88.3%) of all the intracranial tumors diagnosed within the study period were supratentorial. However, among children less than 20 years of age diagnosed with ICT within the study period, majority (61.3%) had the tumor infratentorially located, whereas only 5.3% of the intracranial tumors diagnosed in adults were infratentorially located. The sellar/suprasellar region was the commonest location among the supratentorial tumors diagnosed in children less than 20 years of age, with pilocytic astrocytoma (2 cases), craniopharyngioma (2 cases), pleomorphic xanthoastrocytoma (1 case) and meningioma (1 case) being the diagnosis. (Table 8).

**Table 8:** Locations of some of the intracranial tumors seen within the study period - between 1^st^ of January, 2008 and 31^st^ of December, 2017 at LUTH. Note that the locations of some of the tumors could not be obtained due to loss of clinical information from the manual medical records of the hospital

Intracranial Tumor	Location/Region	Number of Cases
**Meningioma**	Frontal lobe Frontoparietal lobe Temporal lobe Temperoparietal lobe Cerebellum Foramen magnum/Spinal/paraspinal Parasagittal/parafalcine Olfactory groove Covexity Occipital lobe Suprasellar Sphenoidal wing Cerebellopontine angle Parietal lobe Parietal occipital lobe	11 5 5 5 1 6 6 8 3 2 4 8 3 3 2
Pilocytic astrocytoma	Left optic nerve Posterior cranial fossa/cerebellum Suprasellar	1 7 2
Pleomorphic xanthoastrocytoma	Left temporal lobe Suprasellar region	1 1
Diffuse astrocytoma	Frontal lobe Lateral wall of the right ventricle Cerebellopontine angle	1 1 1
Anaplastic astrocytoma	Tempoparietal lobe Left temporal lobe Parietoccipital lobe	1 1 1
Glioblastoms NOS	Occipital lobe Temporal lobe Parietotemporal lobe Frontal lobe Frontoparietal lobe Frontotemporal lobe Suprasellar Foramen magnum	1 3 1 3 1 1 1 2
Pituitary adenoma	Sellar/Suprasellar mass	78
Crainopharyngioma	Sellar/Suprasellar mass	13
Ependymoma	Posterior cranial fossa	4
Medulloblastoma	Cerebellum/Posterior cranial fossa	6
Schwannoma	Cerebellopontine angle	3
Malignant peripheral nerve sheath tumor	Temporal lobe	1
Pinealoblastoma	Pineal gland	1
Oligodendroglioma	Parietal lobe	1
Choroid plexus papilloma	Interventricular region	1

Most of the meningioma cases diagnosed within the study period occurred at the frontal lobe (eleven cases). This is followed by the olfactory groove and sphenoidal wing (eight meningioma cases occurred in each of these locations), then the parafalcine region (six meningioma cases occurred in this location). The cerebellum is the least common area where meningioma occurred, with only one case of meningioma reported in this location (Table 8)

Most of the diffuse astrocytoma occurred at the frontal lobe, while anaplastic astrocytoma cases were mostly located at the tempoparietal region. Glioblastoma (NOS) cases occurred mostly in the temporal lobe followed by the frontal lobe. (Table 8).

The cases of Pilocytic astrocytoma, medulloblastoma, and ependymoma diagnosed within the study period were mostly located within theosterior cranial fossa. (Table 8)

The commonest suprasellar mass was pituitary adenoma (76 cases). This was followed by craniopharyngioma (13 cases). (Table 8).

The most diagnosed tumor of the cranial nerve was schwannoma (3 cases) and all of them were located at the cerebellopontine angle. (Table 8)

## Discussion

From this study, except for a drop in the number of ICTs diagnosed in the year-2014, there was an increment in the frequency of diagnosis of intracranial tumors at LUTH during the decade studied. The reduction in the number of diagnosed intracranial tumors at LUTH in 2014 can be explained by the protracted strike action by the local resident doctors and the Ebola disease epidemic in Lagos, Nigeria that occurred in Lagos, between June and August, 2014. The increasing frequency of diagnosis of ICTs in our environment (Lagos, Nigeria) over the years can be attributed to the increase in diagnostic expertise and the availability and increased use of neuroimaging studies including CT and MRI scans. More so, the increasing public awareness of brain tumors and an increasing life expectancy in Nigeria may have contributed to the rising number of diagnosed ICTs in Nigeria.

The result of this index study, like many other studies done in Nigeria and across the globe, shows that the commonest intracranial neoplasm is meningioma..^1^,^3^,^8^,^9^,^10^,^15^,^19^,^21^,^23^ However, unlike studies done outside Africa, where gliomas ranked second in incidence tomeningiomas, pituitary adenomas was the second most frequent ICT in the index study.^8^,^9^,^10^,^15^ The finding of this study is similar to some other local studies in Nigeria, including studies done by Ohaegbulam *et al.*, Jibrin *et al.*, Igun *et al.* and Idowu *et al.*, that showed an overall increased rate of occurrence of pituitary adenomas among the intracranial tumors, especially when compared to gliomas.^17^,^18^,^20^,^22^ (Table 2) This is in contrast with the global reports, especially from the Western world and Asia, in that both meningioma and gliomas remain relatively more common than pituitary adenomas (Table 1).^6^–^16^

Potential etiological factors to explain this geographical difference, including genetic predisposition, infectious agents, radiation, etc., requires investigation.^5^ The discrepancy in the pattern of occurrence of ICTs in Nigeria when compared with Caucasians may also be attributed to the poor availability of quality health services and poor health seeking behaviour of its citizens, as most people in Nigeria seek for treatment only when there are complications. However, based on evidence from epidemiological research work, some authors have advised cautious interpretation of geographical variations in occurrence of intracranial tumors, since unlike other neoplasms, the criteria and registration of brain tumors is not always consistent.^5^

Our study showed that pilocytic astrocytoma (WHO grade I) and gliobastoma NOS (WHO grade III) were the most frequent astrocytic tumors diagnosed within the study period, each constituted 33.3% of all the astrocytic tumors. This finding is slightly different from that of Malami *et al.* (2019, Sokoto, Nigeria), which showed pilocytic astrocytoma (WHO grade I) as the most diagnosed astrocytic tumors (72.8%), and glioblastoma NOS (WHO grade IV) as the least diagnosed astrocytic tumors (2.0%).^23^

Grade I meningioma constituted 94%, grade II meningioma constituted 6%, while grade III meningioma constituted 0% of all the meningioma cases reported in the index study. These findings are similar to that of Malami et al. (2019, Sokoto, Nigeria), where a similar proportion of the different grades of meningioma (grade I=95%, grade II=6% and grade III=0%) were observed.23 However meningothelial meningioma (WHO grade I) was the most frequent histological variant (55%) in the index study, whereas psammomatous meningioma (WHO grade I) was the most frequent histological variant (75.5%) in the studdone by Malami *et al.*^23^

This study also shows that lymphomas and tumors of the cranial nerves including schwannoma are uncommon in our environment unlike other parts of the world.^8^,^9^,^12^,^13^,^15^ Similar observation was made by Ndubuisi *et al.*^21^

Result of most studies, including those done in Nigeria and globally, showed equal rate of occurrence of intracranial tumors in both genders or a slight male preponderance; many reported a male to female ratio of 1:1.^6^,^7^,^14^,^18^,^19^,^20^,^21^,^22^ However, our study showed a slight female preponderance in the rate of occurrence of intracranial tumors with male to female ratio of 1:1.3. More so, most studies, including the index one, showed a rather increased frequency of occurrence of meningioma in females.^6^,^7^,^14^,^22^

The median age of occurrence of intracranial tumors in the present study was 40.50 years (IQR=30.3), mean age was 37.0 years, and the peak age of occurrence of intracranial tumors in this study was between 41 to 50 years of age (23.7% of the total intracranial tumors). This result is similar to that of Jibrin *et al.* (Abuja, Nigeria, 2018) where the peak age of occurrence of intracranial tumors was noted to be in patients between 40 to 49 years of age, and the mean age of occurrence of ICT was 35±17.1 years.19 Other studies, including local and global studies, showed the mean of occurrence of ICT to be between 26.72 years and 59.18 years.^6^,^14^, ^15^,^19^,^20^ However, our finding is different from that of Ohaegbulam *et al.*(1980, Enugu, Nigeria) , Ndubuisi et al. (2017,Enugu,Nigeria), and Idowu *et al.* (2007, Ibadan, Nigeria) which showed the highest number of intracranial tumors to occur in the first two decades of life and in the sixth decades in their respective studies.^17^,^21^,^22^

Among the pediatric age group, pilocytic astrocytoma was the most common ICT in the index study. This finding is similar to other studies done in Nigeria including studies by Ohaegbulam *et al.*, Olosode *et al.*, Jibrin *et al.* and Ogun *et al.*^17^,^18^,^19^,^25^

There was no primary intracranial tumor of germ cell origin diagnosed among the pediatric age group during the study period. Similar studies done in Nigeria including studies by Olosode et al. and Idowu et al. also reported low rate of occurrence of primary intracranial germ cell tumors.^18^,^22^ This shows that intracranial germ cell tumors are uncommon in our environment when compared to some Asian countries including Japan where there are reports of an increased rate of occurrence of primary intracranial germ cell tumors in children.^15^,^26^

Metastatic intracranial tumors constituted only 1.4 % (5 cases) of all the ICTs studied here with most (4 cases, 80%) of them being carcinomas and one an osteosarcoma. This finding is in keeping with that of Aryal *et al.* who reported eight (14%) of the 57 studied ICTs to be metastatic with seven of them (87.5%) being adenocarcinomas.^7^

Majority (88.3%) of all the intracranial tumors diagnosed within the study period were supratentorial, with the most (94.7%) of the adulthood ICTs being supratentorial and most (61.3%) of the childhood ICTs being infratentorial. This is in keeping with the findings of other study by Idowu et al., where the supratentorial tumors constituted the majority (72.6%) of the ICT studied, with most (57.1%) of the childhood ICTs also being infratentorially located and most (90.1%) of the ICT in adult being supratentorially located.22 This finding is also in concordance with that of Ohaegbulam *et al.*, who reported 95.8% (46 out of the 48 cases) of the ICTs to be supratentorially located.^17^ Additionally, Ohaegbulam *et al.* reported one case of parasellar juvenile astrocytoma, whereas three cases of juvenile astrocytoma (two pilocytic astrocytoma and one pleomorphic xanthoastrocytoma) were reported here. 17 Despite the limitations that exist in the accurate collation of epidemiological data on intracranial tumors in Nigeria, the similarity of the pattern of occurrence of intracranial tumors in various part of Nigeria points to the possibility of a genetic or environmental undertone to its etiology.

The result of the study is similar to other studies done in Nigeria that showed a higher frequency of occurrence of meningioma and pituitary adenoma when compared to gliomas. This is however different from studies done in Caucasians which showed a higher frequency of occurrence of gliomas when compared to pituitary adenoma. This shows that though the rate of occurrence of intracranial neoplasm in our environment, Lagos, Nigeria, is similar to the rate of occurrence worldwide, the pattern of occurrence of different histological types of intracranial neoplasms is different.

## Figures and Tables

**Figure 3:**
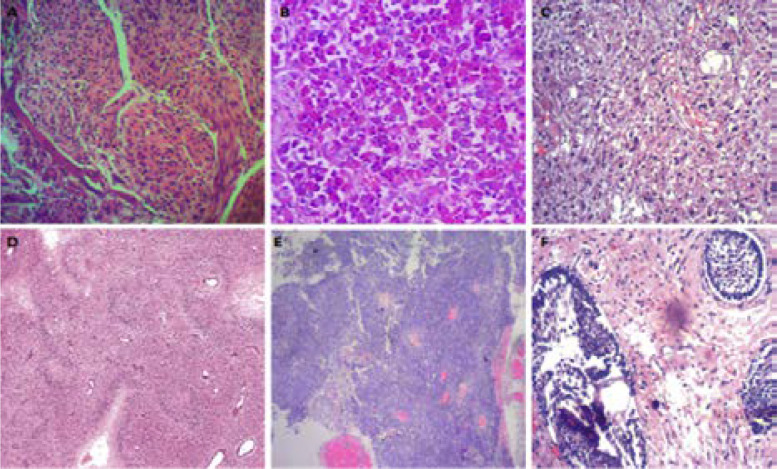
Histologic photomicrographs (Hematoxylin & Eosin stain) of some of the intracranial tumors diagnosed within the study period. A. Meningothelial meningioms (x100 magnification), B. Pituitary adenoma (x100 magnification), C. Pilocytic astrocytoma (x 100 magnification), D. Glioblastoma NOS (x40 magnification), E. Ependymoma (x 40 magnification), F. Craniopharyngioma (x100 magnification).
